# Protein Kinase CK2 Subunits Differentially Perturb the Adhesion and Migration of GN11 Cells: A Model of Immature Migrating Neurons

**DOI:** 10.3390/ijms20235951

**Published:** 2019-11-26

**Authors:** Antonella Lettieri, Christian Borgo, Luca Zanieri, Claudio D’Amore, Roberto Oleari, Alyssa Paganoni, Lorenzo A. Pinna, Anna Cariboni, Mauro Salvi

**Affiliations:** 1 Department of Pharmacological and Biomolecular Sciences, University of Milan, Via Balzaretti 9, 20133 Milan, Italy; antonella.lettieri@hotmail.it (A.L.); luca.zanieri@crick.ac.uk (L.Z.); roberto.oleari@unimi.it (R.O.); alyssa.paganoni@unimi.it (A.P.); 2 Department of Biomedical Sciences, University of Padova, Via U. Bassi 58/B, 35131 Padova, Italy; christian.borgo@unipd.it (C.B.); claudio.damore@unipd.it (C.D.); 3 CNR Institute of Neurosciences, Via U. Bassi 58/B, 35131 Padova, Italy; lorenzo.pinna@unipd.it

**Keywords:** CK2 kinase, neuronal migration, cell adhesion, signaling pathways, microfilaments

## Abstract

Protein kinase CK2 (CK2) is a highly conserved and ubiquitous kinase is involved in crucial biological processes, including proliferation, migration, and differentiation. CK2 holoenzyme is a tetramer composed by two catalytically active (α/α’) and two regulatory (β) subunits and exerts its function on a broad range of targets. In the brain, it regulates different steps of neurodevelopment, such as neural differentiation, neuritogenesis, and synaptic plasticity. Interestingly, CK2 mutations have been recently linked to neurodevelopmental disorders; however, the functional requirements of the individual CK2 subunits in neurodevelopment have not been yet investigated. Here, we disclose the role of CK2 on the migration and adhesion properties of GN11 cells, an established model of mouse immortalized neurons, by different in vitro experimental approaches. Specifically, the cellular requirement of this kinase has been assessed pharmacologically and genetically by exploiting CK2 inhibitors and by generating subunit-specific CK2 knockout GN11 cells (with a CRISPR/Cas9-based approach). We show that CK2α’ subunit has a primary role in increasing cell adhesion and reducing migration properties of GN11 cells by activating the Akt-GSK3β axis, whereas CK2α subunit is dispensable. Further, the knockout of the CK2β regulatory subunits counteracts cell migration, inducing dramatic alterations in the cytoskeleton not observed in CK2α’ knockout cells. Collectively taken, our data support the view that the individual subunits of CK2 play different roles in cell migration and adhesion properties of GN11 cells, supporting independent roles of the different subunits in these processes.

## 1. Introduction

Protein kinase CK2 (CK2) is a master serine–threonine kinase that phosphorylates hundreds of targets [1,2], controlling a wide range of biological cell processes, including proliferation, survival, cell death, differentiation, and migration [3,4,5]. CK2 is generally present as a holoenzyme composed of two catalytic subunits, α and/or α′, and two regulatory β subunits, which combine to form αα′ββ, ααββ, or α′α′ββ heterotetramer, reminiscent of the classical tetrameric structure *of* protein kinase A (PKA). Despite such a similarity, however, both catalytic subunits are active in vitro independent of their association to the β subunits [6]. Nevertheless, the phosphorylation of many typical CK2 targets, such as S129-Akt, S13-Cdc37, and S529-NF-kBp65, is substantially increased by CK2β [7,8]. This suggests that regulatory subunits control the substrate-specific targeting of catalytic subunits. In humans*, CSNK2A1* (CK2α) and *CSNK2A2* (CK2α’) genes encode for the two catalytic proteins, while *CSNK2B* (CK2β) encodes for the regulatory β subunit. Although very similar in the N-terminal region (90% sequence homology), the two catalytic subunits display C-terminal differences that could account for distinct functions in vivo.

The physiological relevance of the different isoforms has been first disclosed by studies on knockout (KO) mice, showing that CK2α is essential for embryos’ growth, with mice dying at early development stages due to cardiac and neural tube defects [9]. Instead, CK2α’ KO mice, although viable, are sterile due to spermatogenesis defects [10], suggesting that CK2α cannot replace all the biological functions of the CK2α’ subunit. CK2β null mice are also not viable, while CK2β heterozygous mice are normal, although they sire offspring at a ratio lower than expected [11]. This implies that at least one regulatory subunit is required for exploitation of the CK2 biological function

Available in vitro studies regarding CK2’s role in cell migration have mainly been focused on tumorigenesis and cancer progression. Some of these works showed that the treatment of different cancer cell lines with specific CK2 inhibitors can delay cell migration [12,13,14,15]. Similarly, siRNA-mediated knockdown of CK2α subunit is sufficient to inhibit the migration of human liver carcinoma HEPG2 [16] and mouse BV-2 microglia cells [17]. Further, the downregulation of CK2α and CK2β via siRNAs inhibits the migration of human laryngeal squamous carcinoma cell line in a wound healing assay, while CK2α’ targeting was ineffective, thus supporting different roles for the two catalytic subunits [18].

CK2 is expressed and constitutively active in the adult mouse brain, with levels of CK2α’ subunit higher in the cortex and hippocampus and lower in the striatum compared to CK2α [19,20,21]. Interestingly, mutations in *CSNK2A1* and *CSNK2B* have been found in patients affected by neurodevelopmental disorders (NDDs), which combine intellectual disability, autism spectrum disorder, and general developmental delay [22,23,24,25,26]. NDDs are mainly caused by defective patterning and/or migration of neurons, which are essential biological processes for proper brain development [27]. Yet, the functional requirement of CK2 in neuronal migration is not known, nor has it been previously attempted to generate stable CK2 KO neuronal lines carrying specific deletions of the single CK2 subunits.

Here, we took advantage of GN11 cells, a model of immature migrating neurons, to study the effects of CK2 on migration and adhesion, by combining pharmacological and genome-editing KO approaches.

First, we studied the role of CK2 in GN11 cells by using two different and structurally unrelated CK2 inhibitors. Then, we dissected the specific functions of each CK2 subunit by generating isoform-specific CK2 KO GN11 cell lines. These experiments highlighted the primary role of CK2α’ subunit in the control of cell migration, whereas the other catalytic subunit (CK2α) is dispensable. We have also shown that the regulatory CK2β subunits are essential for GN11 migration and their deletion induces deep changes in cytoskeletal structures that totally prevent cell migration. Lastly, we dissected the signaling pathways underlying the differences in adhesion and migration between the different KO cell lines, disclosing alteration in the activation of paxillin and Akt.

## 2. Results

### 2.1. Pharmacological Inhibition of CK2 Impairs GN11 Neuron Migration

CK2 regulates the migration of different type of mammalian cells [12,13,14,15,16,17,18] but little is known about its role in neuronal migration. Here, we studied the role of CK2 in a cell model of immortalized immature neurons, GN11 cells [28], that retain migratory activity in vitro.

**Figure 1:**
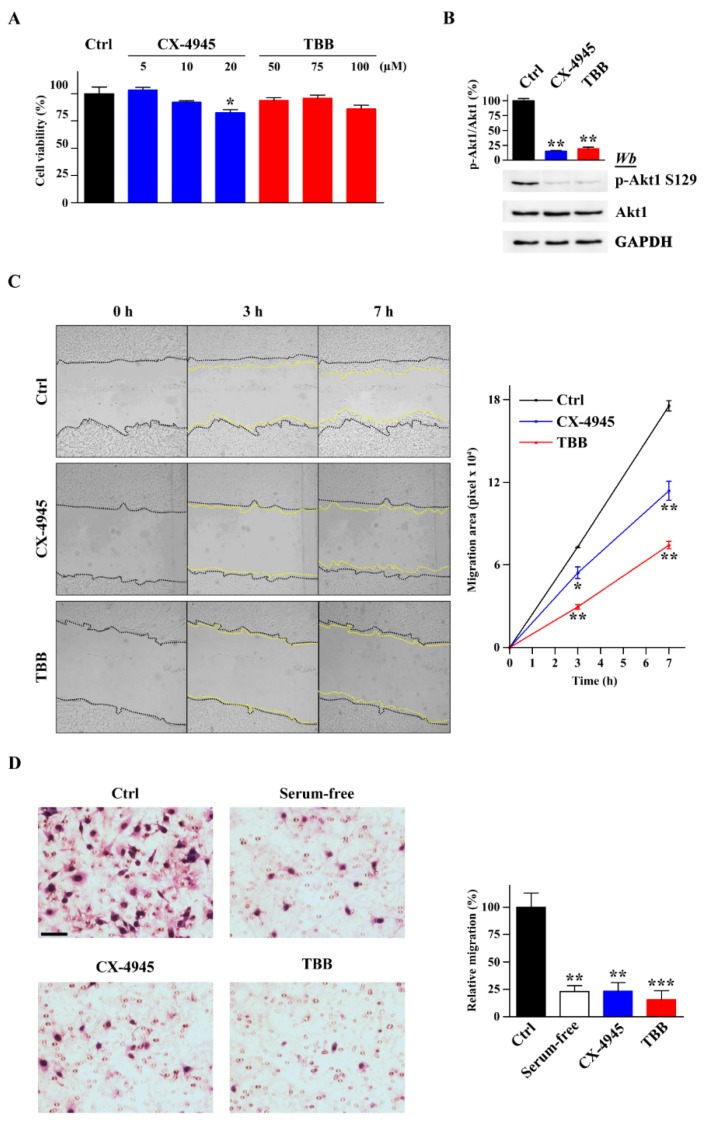
Effect of non-toxic doses of CK2 inhibitors on GN11 cell migration. (**A**) GN11 cell viability was tested by MTT assay after 24-h treatment with different doses of TBB or CX-4945 inhibitors and expressed as a percentage of the control (Ctrl) (*n* = 3). (**B**) Endogenous CK2 activity (*n* = 3) in GN11 cells treated with non-toxic doses of inhibitors (10 µM CX-4945 and 75 µM TBB). Both inhibitors strongly prevented the phosphorylation of CK2-specific target Akt1-S129. (**C**) Scratch assay of GN11 cells treated with 10 µM CX-4945, 75 µM TBB, or vehicle for 24 h (*n* = 3). Representative images for each condition are shown on the left. Quantification of cell migration is reported on the right as the area covered by cells after 3 or 7 h. (**D**) Boyden chamber assay of GN11 cells pre-treated with 10 µM CX-4945, 75 µM TBB, or vehicle for 30 min (*n* = 3). Representative images for each condition are shown on the left. Quantification of migrated cells is represented as the relative migration expressed in percentage; WT cells were used as the reference. Data are shown as mean ± SEM.; * *p* < 0.05, ** *p* < 0.01, *** *p* < 0.001 (one-way ANOVA).

For this purpose, we performed scratch and Boyden chamber assays to measure the chemokinetic and chemotactic properties of migrating cells, respectively, in the presence or absence of two structurally different CK2 kinase inhibitors: CX-4945 (5-((3-chlorophenyl)amino)benzo[c][2,6]naphthyridine-8-carboxylic acid) and TBB (4,5,6,7-tetrabromobenzotriazole). A dose–response curve for each inhibitor was generated in order to select non-toxic concentrations that did not affect cell proliferation in a 24-h assay (Figure 1A). At the two concentrations chosen (10 µM CX-4945 and 75 µM TBB), both inhibitors strongly prevented endogenous CK2 activity, as shown by the robust decrease (more than 75%) in the phosphorylation levels of the CK2-specific target Akt1-S129 [29] (Figure 1B). CK2 inhibitors strongly impaired GN11 cell migration in both scratch (Figure 1C) and Boyden chamber assays (Figure 1D), consistent with an important role for CK2 activity in GN11 cells’ migration.

### 2.2. The Function of Single CK2 Subunits Is Effectively Abolished in GN11 Cells via CRISPR-Cas9 Knockout

**Figure 2:**
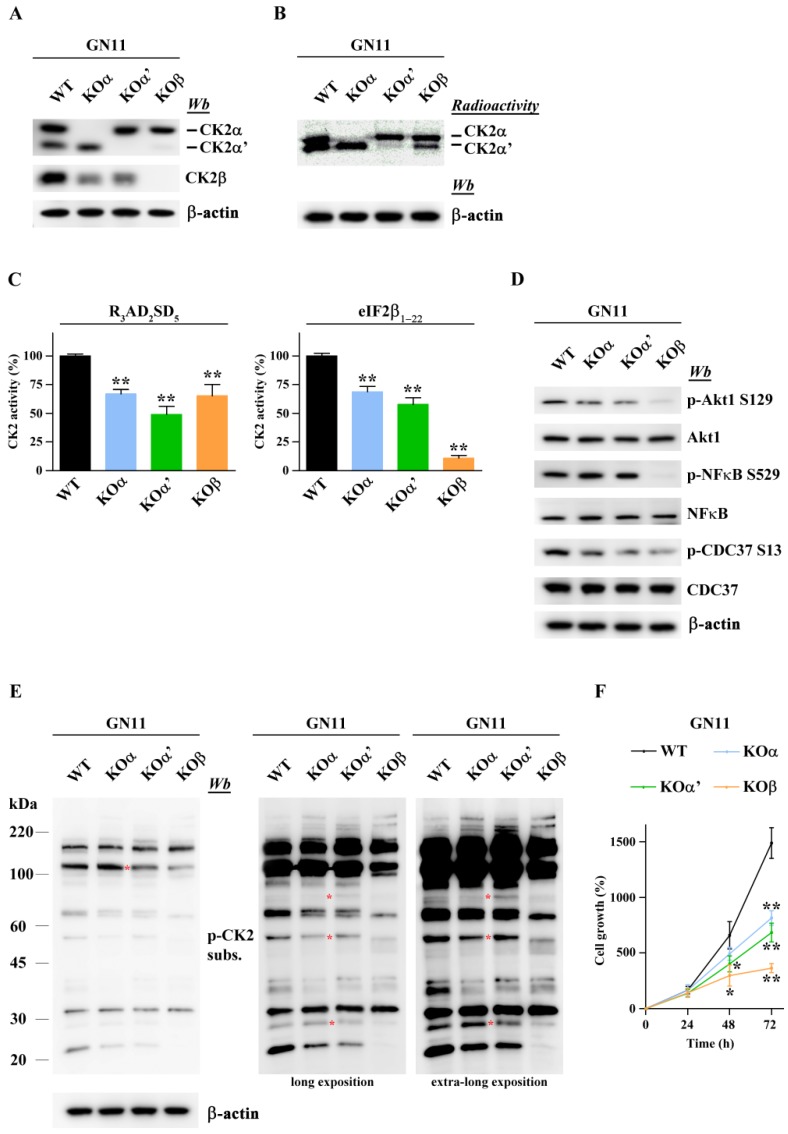
Characterization of CK2 subunit-specific knockout of GN11 cell lines. (**A**) Western blotting analysis confirmed the decreased expression of each subunit in the corresponding CK2 GN11 KO cell line; β-actin was used as a loading control. (**B**) CK2α/α’ activity revealed by in-gel kinase assay. The bands indicate the ^33^P-phosphorylation of β-casein embedded in gel by CK2α and/or CK2α’ of each KO clone. β-actin was used as a loading control. (**C**) CK2 activity was tested in a phosphorylation medium containing lysate proteins, [γ^33^P]ATP, and the substrate peptides RRRADDSDDDDD (R_3_AD_2_SD_5_) or MSGDEMIFDPTMSKKKKKKKKP (eIF2β_1-22_). Kinase activity is expressed as a percentage of the activity of the KO clones compared to the WT (*n* = 3). (**D**,**E**) Phosphorylation of CK2 substrates in WT and CK2 KO GN11 cells**.** Western blot analysis using specific antibodies (**D**) and a general p-CK2 substrates antibody (**E**). Red asterisk indicates bands of protein differentially phosphorylated between KOα and/or KOα’ cells (**E**). β-actin was used as a loading control. (**F**) Cell growth curves were obtained by using the MTT assay. For each cell line, data are expressed at each time point (24, 48, and 72 h) as a percentage of the time 0 (*n* = 3). Data are shown as mean ± SEM.; * *p* < 0.05, ** *p* < 0.01 (one-way ANOVA).

CK2 holoenzyme is composed of two catalytic and two regulatory subunits [3]. To dissect the specific role of each CK2 subunit, three different stable KO GN11 cell lines, each one carrying a deletion in a single CK2 subunit, were generated by applying CRISPR-Cas9 technology. Specifically, we obtained KO cell lines (KOα, KOα’, and KOβ) for CK2α, CK2α’, and CK2β subunits, respectively. The complete lack of each specific CK2 subunit was confirmed by western blot (Figure 2A) and by in gel kinase assay (Figure 2B). The absence of each catalytic subunit, α or α’, also induced a decrease in CK2β levels (Figure 2A), suggesting the instability of β subunits and their subsequent rapid degradation when not incorporated into the CK2 holoenzyme complex, as previously described in the C2C12 cell line [7]. Finally, KOβ cells showed a significant decrease of CK2α’ expression, a regulation that takes place at the transcriptional level as previously demonstrated [7].

The contribution of each catalytic subunit to the overall CK2 activity was quantified by measuring the phosphorylation of two specific substrates: RRRADDSDDDDD (R3AD2SD5), a peptide phosphorylated both by isolated catalytic subunits and tetrameric CK2 isoforms, and MSGDEMIFDPTMSKKKKKKKKP (eIF2β_1–22_), a peptide phosphorylated only by the tetrameric form of CK2 [30]. This analysis showed that CK2 activity was significantly reduced in all KO cell lines compared to WT (Figure 2C).

The two catalytic subunits include a very similar catalytic domain but different C-terminal sequences that could account for isoform-specific targeting. To study the specific role of the two catalytic subunits in phosphorylating endogenous substrates, we evaluated the ability of the three KO cell lines to phosphorylate typical CK2 targets, such as Akt1 S129, CDC37 S13, and NFκB S529, by using phosphospecific antibodies and western blot analysis. Phosphorylation levels of Akt S129 and CDC37 S13 were reduced both in CK2α and CK2α’ KO cells compared to WT GN11 cells (Figure 2D), suggesting that these substrates can be phosphorylated by both catalytic subunits (Figure 2D). In contrast, the phosphorylation levels of S529 NFκB were not significantly reduced by knocking out either CK2α or CK2α’; on the other hand, the phosphorylation levels of the three targets were strongly reduced in the KOβ cells (Figure 2D).

To have an overall view of phosphosites that can be affected by CK2 subunit-specific loss, we used antibody-recognizing phosphosites, which contain the CK2 consensus sequence (pS/pT)DXE, whose specificity has been previously demonstrated [7]. Figure 2E shows that there are phosphosites sensitive to the lack of one but not the other catalytic subunit (see bands marked with an asterisk in Figure 2E), denoting that these two catalytic isoforms may regulate different signal transduction pathways. Notably, the highest decrease in the phosphorylation rate was observed in KOβ cells, consistent with the concept that phosphorylation of the majority of CK2 substrates is optimally catalyzed by CK2 holoenzyme [8,31].

### 2.3. Differential Inactivation of CK2 Subunits Results in Reduced Proliferation and Different Migratory Activity of GN11 Cells

Because CK2 is involved in cell proliferation, we first compared the proliferation rate of CK2 KO lines versus WT cells, with MTT. All three KO cell lines showed a significant reduction in proliferation after 48 and 72 h, with a similar decrease in the proliferation rate for KOα and KOα’ and a more pronounced decrease for the KOβ cell line (Figure 2F). This suggests that the two catalytic subunits are both required to sustain cell proliferation and do not compensate each other, and that the regulatory subunits also contribute to proliferation processes.

**Figure 3:**
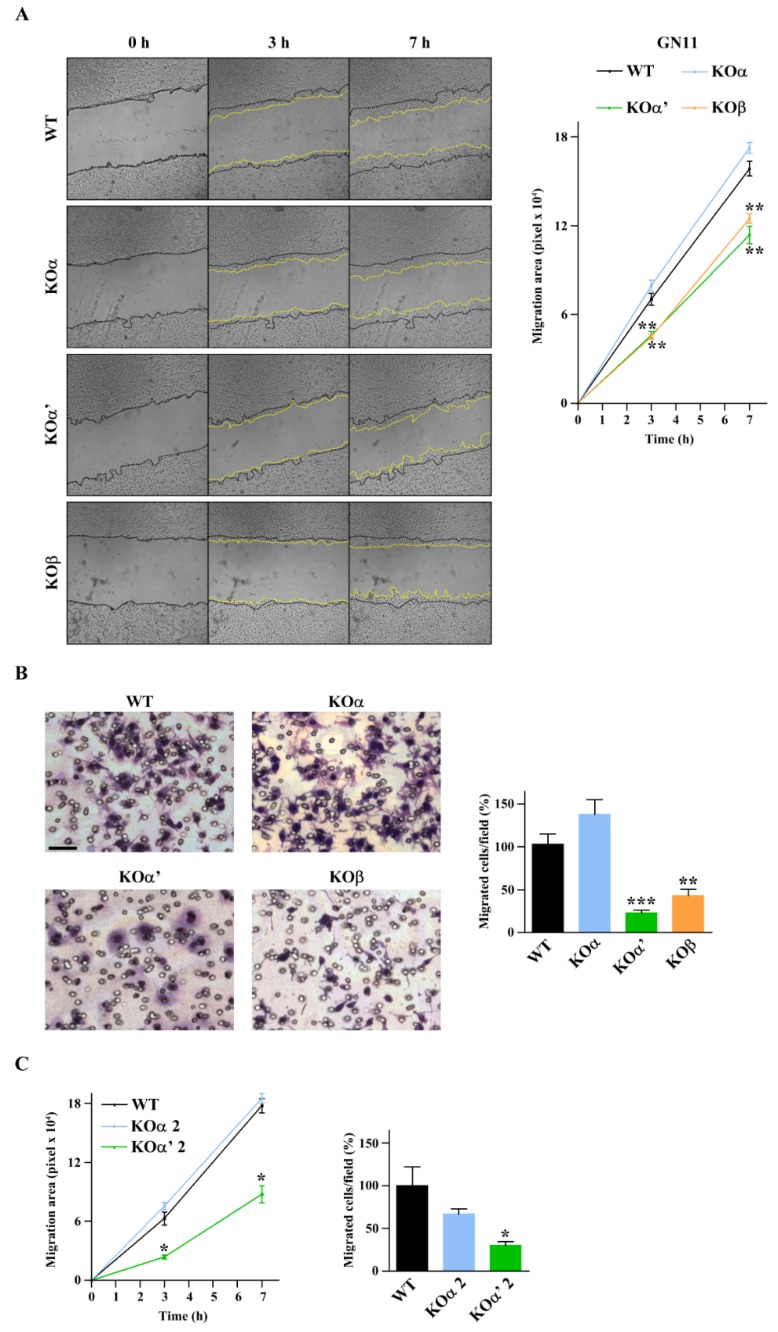
Loss of CK2 α’ and β but not α subunits affects GN11 cell migration. (**A**) Scratch assay of GN11 cells of indicated genotypes. Representative images for each condition are shown on the left. Quantification of cell migration is reported on the right as covered by cells after 3 or 7 h (*n* = 3). (**B**) Boyden chamber assay of GN11 cells of indicated genotypes. Representative images for each condition are shown on the left. Quantification of migrated cells is represented as relative migration expressed in percentage; WT cells were used as a reference (*n* = 3). (**C**) Quantification of migration ability of a different clone of KOα and KOα’, named KOα 2 and KOα’ 2, respectively, after scratch assay (*n* = 3) (left) and Boyden chamber assay (*n* = 3) (right). Data are shown as mean ± SEM.; * *p* < 0.05, ** *p* < 0.01, *** *p* < 0.001 (one-way ANOVA).

Migration properties of the different CK2 subunit-specific KO cell lines were first tested by an in vitro scratch assay, by measuring the surface occupied by wild type and CK2 KO cells after 3 and 7 h from the scratch. As shown in Figure 3A, the absence of CK2α did not significantly affect cell migration. On the contrary, the lack of either CK2α’ or CK2β significantly delayed wound closure (Figure 3A). Boyden assays were then performed to evaluate whether chemotaxis was also impaired. As shown in Figure 3B, KOα’ and KOβ cells migrated significantly less as compared to WT GN11 cells, while the ablation of CK2α did not affect the chemomigration of GN11 cells.

Since CK2α is considered the main catalytic isoform with a primary role in cell migration [12,13,14,15,16,17,18], our results were unexpected. Thus, to exclude that the results observed could be due to off-target effects of the CRISPR/Cas9 technique, we generated two additional KO lines for CK2 α and α’ subunits, respectively, called KOα 2 and KOα’ 2, by designing two RNA-guided sequences (sgRNAs) targeted to different sequences of *CK2α* and *CK2α’* genes. Like the original clones, each of the new clones displayed the same migratory behavior: CK2α’ deletion prevented GN11 cell migration both in scratch and Boyden assay experiments, whereas CK2α deletion was ineffective (Figure 3C). Altogether, these data outline a primary role of the tetrameric CK2 holoenzyme and a specific role of the CK2α’ catalytic subunit in the migration properties of GN11 cells.

### 2.4. CK2 α’ and β Subunit Knockout Cell Lines Display a Different Cytoskeleton Organization Compared to Wild-Type GN11 Cells.

**Figure 4:**
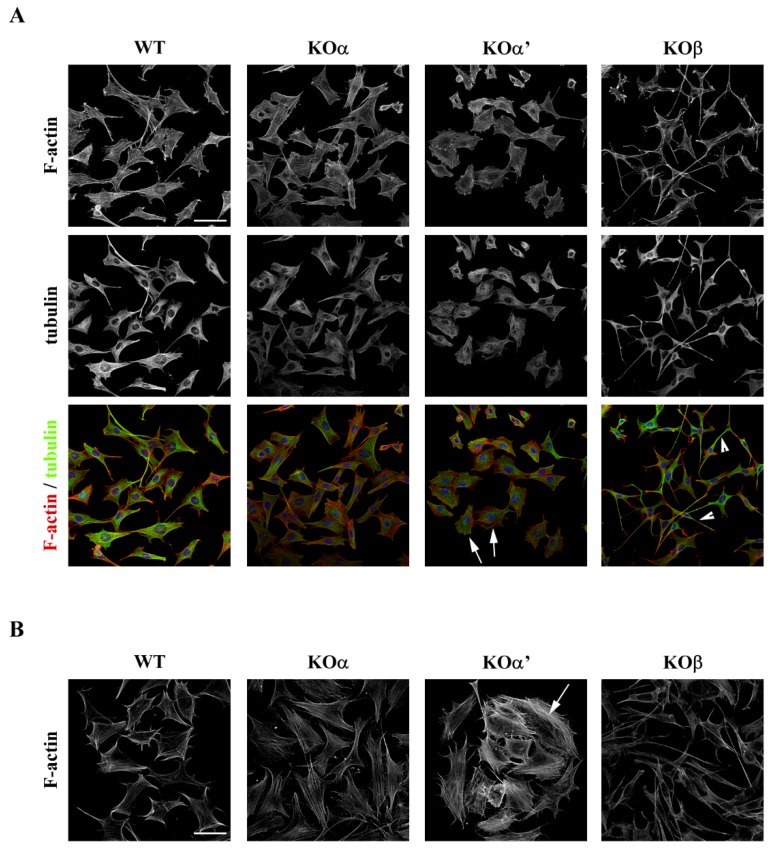
Loss of CK2 α’ and β subunits but not α subunit alters cytoskeleton organization. (**A**) GN11 cells of indicated genotypes were immunolabelled for F-actin (red) and α-tubulin (green) to reveal microfilament and microtubule organization, respectively. Single channels are shown as black and white images. Arrows point at examples of KOα’ cells displaying a flat morphology whereas arrowheads point at examples of KOβ displaying a prominent fusiform morphology. (**B**) High magnification images of GN11 cells of the indicated genotypes stained for F-actin. An arrow indicates increased stress fibers in KOα’ cells. Actin depolymerization in KOβ cells is also shown. Scale bar: 40 um (**A**); 25 um (**B**).

To compare the effects of CK2 inactivation on cell morphology, we studied the cytoskeleton organization of each KO cell line, by performing immunostainings for F-actin and α-tubulin. As shown in Figure 4A, KOα cells appeared similar to WT cells while KOα’ were flatter and less elongated compared to WT cells. In contrast, KOβ cells displayed a smaller cellular body and their axons appeared longer, compared to WT cells. No gross differences were observed in α-tubulin organization between the different genotypes, whereas differences in actin organization were found (Figure 4B). KOα’ cells displayed a larger number of actin stress fibers compared to WT cells while the KOα cells showed an actin organization comparable to WT cells (Figure 4B). KOβ cells did not exhibit proper actin fibers, with a punctate aspect typical of depolymerized actin [32,33]. These results suggest that the inability of KOβ cells to migrate is attributable to severe defects in actin cytoskeletal structures that are required for proper migration [34]. Further, the increased number of stress fibers and surface observed in KOα’ cells may be related to increased adhesion [34,35].

### 2.5. CK2 Knockout Affects GN11 Adhesion and Signal Transduction

**Figure 5:**
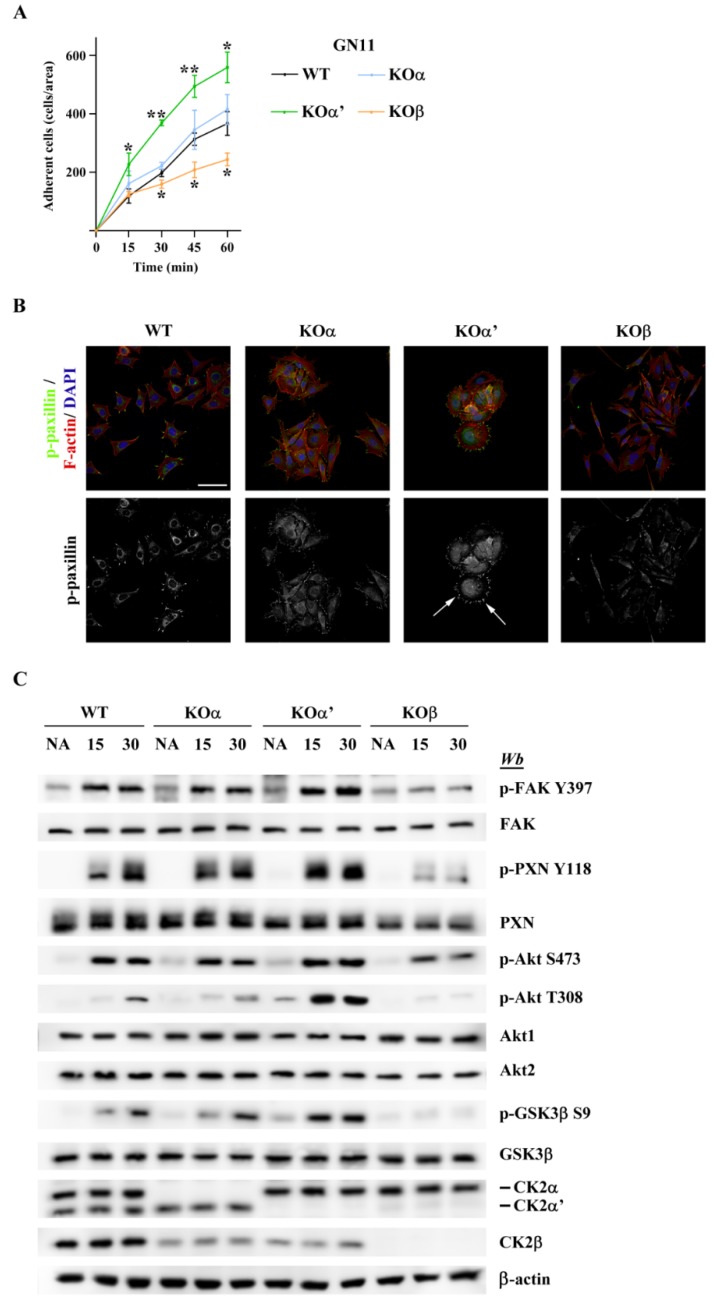
Impaired fibronectin (FN)-induced adhesion of CK2α′ and CK2β KO GN11 cells is due to different signaling pathways. (**A**) FN-mediated adhesion curves of WT and indicated KO GN11 cells were evaluated by direct cell counting at 15, 30, 45, and 60 min. Results of three independent experiments are shown as mean ± SEM.; * *p* < 0.05, ** *p* < 0.01 (one-way ANOVA). (**B**) GN11 cells were allowed to adhere to FN for 60 min and immunostained for p-paxillin (p-PXN, green) and F-actin (red) to reveal focal adhesion sites and F-actin filament, respectively, and DAPI (blue) to stain the nuclei (upper panel). The white arrowheads indicate regions in KOα’ cells that are rich in p-PXN (lower panel). Scale bar, 25 µm. Images shown in the panel are representative of at least three separate experiments. (**C**) WT and indicated KO GN11 cells were serum-starved for 16 h, then detached in serum-free medium (non-adherent, NA), and/or plated on FN for indicated times. Whole cell lysates were analyzed by western blot with indicated antibodies. β-actin was used as loading control. The panel is representative of at least five separate experiments.

A reduced cell motility is generally correlated with reduced adhesion turnover [34,35]. Thus, to verify this hypothesis, we first compared cell adhesion properties of the different cell lines and then we studied the activation of specific adhesion signaling pathways. As shown in Figure 5A, KOα’ cells were more adherent compared to the wild type counterpart after just 15 min of exposure to fibronectin (FN), while KOα cells were similar to WT cells. On the contrary, KOβ cells were less adherent than WT (Figure 5A), consistent with the severe cytoskeleton defects displayed by these cells (Figure 4).

To study the signaling pathways linked to adhesion and migration activated in the different CK2 KO cell lines, we first analyzed paxillin (PXN) and focal adhesion kinase (FAK), two critical regulators of focal adhesion, by immunocytochemistry and by western blotting (Figure 5B,C). The analysis of p-PXN distribution revealed that KOα’ cells were round in shape and displayed stronger phosphorylation of paxillin at their focal adhesion contacts, as compared to WT and KOα cells. Instead, KOβ cells showed low p-paxillin signal intensity (Figure 5B). To better characterize the underlying signaling mechanisms, we compared the phosphorylation extent of p-FAK Y397 and of the Y118 site on the FAK target scaffold protein paxillin, which is important for FAK redistribution to focal adhesion, in all the cell lines (Figure 5C). No differences in phosphorylation levels were observed between KOα and WT cells, in agreement with the outcome of the migration and adhesion assays. Instead, in KOα’ cells, FAK Y397 autophosphorylation levels were higher than that in WT cells (Figure 5C), which positively correlated with its kinase activity [36]. Accordingly, p-PXN Y118 phosphorylation levels were also increased (Figure 5C).

A higher phosphorylation of FAK and paxillin can correlate with increased cell adhesion and reduced cell migration, but also with increased cell migration and increased adhesion turnover [37]. To distinguish between these two alternatives, we analyzed the activation of Akt, a protein kinase that plays a pivotal role in cellular motility and migration [38]. Fully activated Akt requires phosphorylation of both T308 and S473 [39]. Figure 5C shows that Akt is strongly activated in KOα’ cells and this activation correlates with an increase in phosphorylation of the GSK3β S9, which downregulates the kinase. GSK3β is constitutively active and regulates cytoskeletal dynamics in migrating cells by phosphorylating different microtubule-associated proteins [40]. More importantly, GSK3β S9 phosphorylation and inactivation has been implicated in increased cell adhesion and decreased migration [41]. Therefore, the gain in substrate adhesion and the impairment in migration observed in our cellular model could be explained by an increase in focal adhesion activation associated with an inhibition along the Akt–Gsk3β axis.

The same signaling pathways were also analyzed in KOβ cell lines, which displayed reduced cell migration and adhesion compared to WT cells. Accordingly, a strong reduction of FAK and PXN phosphorylation was observed in these cells, thus accounting for reduced substrate-specific adherence. CK2β cells also displayed defective Akt and GSK3β phosphorylation (Figure 5C). Overall, these results support the hypothesis that CK2β may play additional roles outside of the CK2 tetrameric complex.

## 3. Discussion

CK2 is a tetrameric Ser/Thr protein kinase that phosphorylates a plethora of different substrates and participates in numerous cellular physiological and pathological processes, such as proliferation, survival, apoptosis, angiogenesis, cancer progression, DNA damage and repair, ER stress response, carbohydrate metabolism, and brain development [5]. Consistently, CK2 is ubiquitously expressed in the periphery as well as in the brain [42]. Yet, very little is known about the role of CK2 in brain development and specifically in neuron biology.

Recently, mutations in *CSNK2A1* and *CSNK2B* genes, encoding for α catalytic subunit and β regulatory subunits, respectively, have been found in patients with neurodevelopmental disorders (NDDs) [22,23,24,25,26], but the functional role of CK2 has not been elucidated in these works. The term NDDs applies to a broad group of disabilities caused by defective brain development affecting more than 3% of children [43]. Among them, a very wide range of neurological and psychiatric problems that can be clinically and causally different are evident, including rare genetic syndromes, schizophrenia, autism, and epilepsy [44]. Many of these conditions arise from dysfunctional genes, which exert critical roles in brain development and/or function [45], including cell migration. Neuron migration is a critical biological process for proper brain organization during development and several NDDs are due to defective neuron migration and positioning [46]. Thus, the comprehension of molecular pathways that regulate neuronal migration is essential to fully understand the etiology of human NDDs [27].

In this paper, we disclosed a key role of the protein kinase CK2 in controlling the migration and adhesion of an established model of immortalized immature mouse neurons (GN11 cell line [47,48,49]). Using two highly specific and structurally unrelated CK2 inhibitors, we found that CK2 activity is required for the migration of GN11 cells. Moreover, by selectively knocking out each of the three subunits that make up CK2 holoenzyme (by genome editing), we dissected the specific contribution of each of them to GN11 migration, adhesion, and signaling pathway activation. Notably, our single KO cellular models confirm the hypothesis that the two catalytic subunits, even if quite similar, may have different subsets of substrates. Between the two catalytic subunits, we showed that CK2α’ has a primary role in cell migration and adhesion, while CK2α is dispensable. This was clearly an unexpected result, but it demonstrates that the role of the two catalytic subunits could be different according to the cell type and that the critical contribution of the “ugly duckling” CK2α’ should not be overlooked.

Our findings revealed that the deletion of CK2β regulatory subunits also induces a decrease in cell migration; however, the underlying mechanisms are clearly different. In CK2α’ KO cells, we observed a decrease in cell migration combined with an increase in cell adhesion to fibronectin. This has been associated with the activation of focal adhesion molecules, such as FAK and paxillin, and, on the other side, by Akt activation and GSK3β inhibition.

Instead, when we knocked out the regulatory β subunit, GN11 cells displayed a decrease in both cell migration and adhesion compared to WT cells. The reduced adhesion is consistent with a weak activation of FAK/paxillin and Akt. In particular, KOβ cells displayed a general actin depolymerization that clearly correlated with defective migration.

The different behavior between KOβ and KOα’ cells suggests that the role of CK2β may be not just that of a regulatory subunit of the CK2 tetrameric complex. Interestingly, in the last few years, additional roles for the β subunit have been hypothesized. For example, CK2β has been proposed as a binding partner of other kinases, including A-Raf, c-Mos, and Chk1 [50]. This is in agreement with the phosphoproteomic analysis we performed in the CK2β KO C2C12 cell line, where we showed that a large number of phosphosites not conforming to the CK2 consensus are also affected [8]. Moreover, recently, it has been reported that CK2β controls the myogenic commitment of C2C12 cells by regulating MyoD expression independently from catalytic subunits [51]. Thus, further studies will be necessary to shed light on the CK2-independent role of the CK2β subunit for a better understanding of its biological roles.

In summary, our results show that the loss of CK2 α’and β subunits severely disrupts GN11 cell migration and additionally affects cell adhesion, via the activation of different signaling pathways, thus providing the first evidence of CK2 requirement in neuron migration. This may be relevant to the observation that mutations in genes encoding for CK2 subunits have been identified in patients affected by NDDs, supporting the idea that CK2 is indeed required for the proper neuronal migration underlying brain development.

## 4. Material and Methods

### 4.1. Chemicals and Antibodies

[γ-^33^P]ATP was purchased from Hartmann Analytic GmbH (Braunschweig, Germany). Protease inhibitor cocktail was from Calbiochem (Darmstadt, Germany), while phosphatase inhibitor cocktails 2 and 3 were from Sigma-Aldrich (Dorset, UK). RRRADDSDDDDD and MSGDEMIFDPTMSKKKKKKKKP peptides were kindly provided by Prof. Oriano Marin (University of Padova). CX-4945 (5-[(3-Chlorophenyl)amino]-benzo[c]-2,6-naphthyridine-8-carboxylic) was purchased from Glixx Laboratories (South Borough, MA, USA); TBB (4,5,6,7-tetrabromobenzotriazole) was from Selleckchem (Houston, TX, USA). Inhibitor solutions were prepared in DMSO. Anti-CK2β (ab76025), anti-phospho-Akt1 S129 antibodies (ab133458), and anti-phospho-CDC37 S13 (ab108360) were from Abcam (Cambridge, UK). Anti-β-actin antibody (A2228) and anti-α-tubulin antibody (T5168) were purchased from Sigma-Aldrich (Saint Louis, MO, USA). Anti-Akt1 (sc-5298), anti-CDC37 (sc-13129), anti-phospho-NF-kBp65 S529 (sc-101751), anti-NF-kBp65 (sc-109), anti-phosho-FAK Y397 (sc-81493), and anti-FAK (sc-271126) antibodies were from Santa Cruz Biotechnology (Dallas, TX, USA). Anti-phospho-paxillin Y118 (#2541), anti-phospho-Akt S473 (#4060), anti-phospho-Akt T308 (#13038), anti-Akt1 (#75692), anti-Akt2 (#3063), anti-phospho-GSK3β S9 (#9323), and anti-GSK3β (#9832) were from Cell Signaling Technology (Leiden, Netherlands). Anti-paxillin (GTX-125891) was from GeneTex (Irvine, CA, USA). Secondary antibodies towards rabbit and mouse IgG, conjugated to horseradish peroxidase, were from PerkinElmer (Waltham, MA, USA). TRITC-conjugated phalloidin was used to stain F-actin (P1951, Sigma-Aldrich).

### 4.2. Cell Culture

Wild-type or mutant GN11 cells [28] were maintained in DMEM supplemented with 10% FBS, 2 mM l-glutamine, 100 U/mL penicillin and 100 mM streptomycin, in an atmosphere containing 5% CO_2_.

### 4.3. Cell lysis and Western Blotting Analysis

Cells were detached, centrifuged, washed with PBS, and lysed for 20 min (min) on ice in the lysis buffer containing 20 mM Tris–HCl (pH 8.0), 150 mM NaCl, 2 mM EDTA, 2 mM EGTA, 1% Triton X-100 (*v*/*v*), protease inhibitor cocktail Complete (Roche, Basel, Switzerland), and phosphatase inhibitor Cocktail 2 and 3 (Sigma). Cell lysates were centrifuged at 10,000× *g* for 10 min at 4 °C. The supernatant was collected, and protein concentration was determined by the Bradford method. Equal amounts of total protein extracts were loaded on SDS-PAGE, blotted on Immobilon-P membranes (Millipore, Burlington, MA, United States), processed by western blot with the indicated antibody, and detected by chemiluminescence on ImageQuant LAS 500 (GE Healthcare Life Sciences, Chicago, IL, USA).

### 4.4. CRISPR/Cas9-Mediated CK2 Knockout

All-in-one plasmids expressing Cas9-DasherGFP and the sgRNA guide (pD1301-AD: CMV-Cas9-2A-GFP, Cas9-ElecD) to target the specific CK2 subunits were purchased from DNA 2.0, Inc. The two CK2α sgRNA guide sequences are 5′-CCTGGATTATTGTCACAGCA-3′ (n.11) and 5′-CATAATGTCATGATTGATCA-3′ (n.91); the two CK2α’ sgRNA guide sequences are 5′-AACTGGTTCGAAAACTTGGT-3′ (9) and 5′-GTTCACCTCGGCGTAGACCC-3′ (n.49); and the CK2β sgRNA guide sequence is 5′-TCCTGGTTCTGTGGGCTCCG-3′. GN11 cells were cultured in six-well dishes to 70% to 80% confluence. Cells were transfected with 1 μg of plasmid-expressing sgRNA and Cas9-GFP with Lipofectamine 3000, according to the manufacturer’s instructions. Forty-eight hours post-transfection, cells were pelleted in PBS and sorted in 96-well plates using fluorescence-activated cell sorting (FACS) with a FACSAria II cell sorter (BD BioSciences). Single cells were expanded to obtain individual clones. Individual clones were lysed and quantified as described above. The absence of the specific CK2 subunits was verified by western blotting and kinase activity assay.

### 4.5. CK2 Kinase Activity Assay

Proteins from cell lysates (1 μg) were incubated for 10 min at 30 °C in 25 μL of a phosphorylation medium containing 50 mM Tris–HCl (pH 7.5), 100 mM NaCl, 10 mM MgCl_2_, 400 μM synthetic peptide-substrate RRRADDSDDDDD (R_3_AD_2_SD_5_) or MSGDEMIFDPTMSKKKKKKKKP (eIF2β_1-22_), and 50 μM [γ-^33^P]ATP (1000 cpm/pmol). Assays were stopped by absorption onto phosphocellulose filters. Filters were washed four times in 75 mM phosphoric acid and analyzed by a Scintillation Counter (PerkinElmer).

### 4.6. In-Gel Kinase Assay of CK2α/α’

The activity displayed by CK2 subunits were determined by running cell lysates on an 11% SDS-PAGE containing the CK2-substrate β-casein (0.5 mg/mL). After electrophoresis, gel was then washed and incubated in a renaturating buffer, leading to the recovery of the active CK2α/α′ conformation [52]. The activity of CK2α and CK2α′ towards the co-localized β-casein was detected by incubating the gel with the above-described phosphorylation medium containing 10 μM [γ^33^P]ATP. Radioactive ^33^P-β-casein was evidenced by analyzing the dried gel with a Cyclone Plus Storage PhosphorSystem (PerkinElmer).

### 4.7. Proliferation Assay

Cell proliferation was assessed by monitoring the conversion of MTT [3-(4,5-dimethylthiazol-2-yl)2,5-diphenyltetrazolium bromide] to formazan. Briefly, cells were seeded at 5 × 10^4^ and 1 × 10^5^ cells/mL into 96-well plates (100 µL/well) and allowed to adhere for 24 h. Cell growth was assessed by adding 10 μL of MTT solution (5 mg/mL in PBS) to each well for 1 h. Incubation was stopped by the addition of 20 μL of lysis solution at pH 4.7, containing 20% (*w*/*v*) SDS, 50% (*v*/*v*) *N*,*N*-dimethylformamide, 2% (*v*/*v*) acetic acid, and 25 mM HCl. The *A*_590_ of plates was measured using a Titertek Multiskan Plus plate reader (Flow Laboratories). Six replicates of each sample were assayed. The viability of cells was calculated by plotting the absorbance of treated cells against the control cells treated with DMSO after 24 h. The cell growth was evaluated by plotting the absorbance at each time point (24, 48 and 72 h) against the absorbance at time 0 for each cell line.

### 4.8. Immunocytochemistry

Wild-type and CK2KO cell lines were seeded on 13-mm coverslips at the density of 15,000 cells/well. The day after plating, cells were starved for 12 h and fixed with 4% PFA or 100% MeOH depending on the downstream staining. To detect F-actin, cells were fixed in PFA and stained with TRITC-conjugated phalloidin (1:400) for 30 min at 37 °C. To detect α-tubulin, cells were fixed with MeOH and immunolabelled with an anti-mouse α-tubulin (1:500), for 1 h at RT, followed by an Alexa Fluor 488-conjuaged secondary antibody. To detect focal contacts, cells were allowed to adhere to FN for 1 h, fixed with PFA, and immunolabelled with an anti-phospho paxillin antibody (1:100) for 1 h at RT followed by an Alexa Fluor 488-conjugated secondary antibody.

### 4.9. Migration Assays

For the scratch assay, cells were grown to confluence on 30-mm culture dishes. Cells were then scratch wounded using a sterile 200-μL pipette tip, washed twice with phosphate-buffered saline (PBS) to remove suspended cells, and covered with complete medium. For the experiments with inhibitors, cells were pre-treated for 16 h before the wound was performed. The scratch areas of at least 5 fields/group were photographed and recorded at different time points (0, 3, and 7 h). Using the ImageJ image processing program, migration areas (in pixel) were determined from the digital images and calculated as A_0_–A (A_0_, scratch area at time 0 h; A, uncovered area by cells at each time point analyzed).

For the Boyden Chamber assay, sub-confluent cells were collected and resuspended in DMEM 0.1% BSA at a final concentration of 2 × 10^6^ cells/mL and exposed to DMEM *w*/*o* FBS (negative control) or with 10% of FBS (positive control) for 3 h at 37 °C. For the experiments with inhibitors, resuspended cells were pre-treated for 30 min with the vehicle or inhibitor. Unmigrated cells were gently scraped away and migrated cells were fixed and stained as previously described [53,54]. Each condition was performed in quadruplicate in three different independent experiments.

### 4.10. Adhesion Assay and Signal Transduction Pathways Analysis

Serum-starved (16 h) wild-type or mutant GN11 cells were detached with trypsin and resuspended in DMEM serum-free (supplemented with 2 mM L-glutamine, 100 U/mL penicillin, and 100 mM streptomycin). For the cell adhesion evaluation, 2 × 10^5^ cells/dish were seeded in triplicate on a 24-well tissue culture dish coated overnight with 10 μg/mL FN in PBS. After 15, 30, 45, and 60 min, cells were analyzed live with a microscope (Euroteck orma, Sesto San Giovanni, Italy) provided with a DCC1645C camera (ThorLabs, Newton, NJ, USA) and equipped with a 109/0.25 objective. Bright-field images were captured with THORCAM UC480 viewer software (ThorLabs). At each time-point, 5 different fields per well were captured and the number of adherent cells was analyzed by IMAGEJ software. For the cell signaling study, 2 × 10^6^ cells/dish were seeded on a 35-mm tissue culture dish coated overnight with 10 μg/mL FN in PBS for 15 or 30 min. After washing three times with PBS, cells were lysed by scraping in ice-cold lysis buffer. Then, equal amounts of cell lysates were subjected to 9% SDS-PAGE and western blot analysis.

### 4.11. Quantifications and Statistical Analyses

For the scratch assay, the area occupied by migrating cells was measured by using ImageJ software.

For the adhesion assay, four random fields/well for each cell line triplicate were acquired by using a digital camera connected to a Zeiss inverted microscope (Axio Scope) and the number of adherent cells was calculated as the mean of cells/field.

For the Boyden assays, three random fields of stained cells were counted for each well/condition, and the mean number of migrating cells/field for each chemoattractant condition was calculated.

Statistical analysis was performed by one-way ANOVA with post-hoc Tukey’s test. Values are expressed as mean ± SEM.
